# A Live Zebrafish-Based Screening System for Human Nuclear Receptor Ligand and Cofactor Discovery

**DOI:** 10.1371/journal.pone.0009797

**Published:** 2010-03-22

**Authors:** Jens Tiefenbach, Pamela R. Moll, Meryl R. Nelson, Chun Hu, Lilia Baev, Thomas Kislinger, Henry M. Krause

**Affiliations:** 1 Banting and Best Department of Medical Research, The Terrence Donnelly Centre for Cellular and Biomolecular Research (CCBR), University of Toronto, Toronto, Ontario, Canada; 2 InDanio Bioscience Inc., Toronto, Ontario, Canada; 3 Department of Medical Biophysics, University of Toronto, Toronto, Ontario, Canada; 4 Ontario Cancer Institute, University Health Network, Toronto, Ontario, Canada; 5 Campbell Family Cancer Research Institute, Toronto, Ontario, Canada; Ecole Normale Supérieure de Lyon, France

## Abstract

Nuclear receptors (NRs) belong to a superfamily of transcription factors that regulate numerous homeostatic, metabolic and reproductive processes. Taken together with their modulation by small lipophilic molecules, they also represent an important and successful class of drug targets. Although many NRs have been targeted successfully, the majority have not, and one third are still orphans. Here we report the development of an *in vivo* GFP-based reporter system suitable for monitoring NR activities in all cells and tissues using live zebrafish (*Danio rerio*). The human NR fusion proteins used also contain a new affinity tag cassette allowing the purification of receptors with bound molecules from responsive tissues. We show that these constructs 1) respond as expected to endogenous zebrafish hormones and cofactors, 2) facilitate efficient receptor and cofactor purification, 3) respond robustly to NR hormones and drugs and 4) yield readily quantifiable signals. Transgenic lines representing the majority of human NRs have been established and are available for the investigation of tissue- and isoform-specific ligands and cofactors.

## Introduction

Nuclear receptors (NRs) are ligand-activated transcription factors that regulate the expression of specific gene networks by recruiting co-activator or co-repressor complexes. In doing so, they regulate diverse physiological processes such as metabolism, development, growth and reproduction. NRs share a modular structure, which includes highly conserved DNA- and ligand-binding domains (DBDs, LBDs) spaced by a variable hinge region [1], [2]. Their activities are modulated by small hydrophobic compounds, such as steroids, fatty acids, retinoids and thyroid hormones. Ligand binding to the LBD alters its conformation, cofactor binding and/or transcriptional activity [3]. About a third of NRs, however, are referred to as orphan receptors, as the identities of their natural ligands are still unknown.

NR ligands, or drugs that mimic them, have been used to deal with many major and debilitating diseases [4], [5], [6]. However, only a small percentage of NRs have been targeted, and even for these, drugs that act more selectively would provide huge benefits. New drugs capable of modulating orphan NR activities have the potential to control numerous additional disorders such as heart disease, atherosclerosis, metabolic disease, cancer, inflammation, depression and anxiety [7], [8].

Currently used methods to analyze NR activities and to identify their ligands are mainly *in vitro*- or cell culture-based. There are two major drawbacks of these approaches. First, they fail to predict the *in vivo* delivery, stability and specificity of these molecules within the human body. Second, they only assess a single molecular interaction or cell type, while other tissue-specific ligands, cofactors and conditions are ignored. These issues could be greatly reduced by the adoption of *in vivo* screening approaches. However, initial attempts, using β-galactosidase-based reporter systems [9], [10], [11], [12] have proven tedious, due to a requirement for tissue fixation, and in vertebrates, dissection or serial sectioning. More recently, the adoption of fluorescent reporters, such as the Green Fluorescent Protein (GFP), has allowed the monitoring of live tissues [9], [10], [11], [13], [14]. By combining this use of flurescent reporters, together with a transparent and ex-utero developing organism such as the zebrafish, has now made it possible to design fluorescent reporter systems in live vertebrates.

Zebrafish constitute a powerful animal model, due largely to their small size, optical clarity, ex-utero development, fecundity, rapid development and large arsenal of readily available genetic tools. Importantly, embryos, hatchlings and mature fish readily absorb/intake compounds from their aqueous environment and are DMSO tolerant [15], [16]. Vertebrate NRs, cofactors and ligands are highly related, such that in most examined cases, mutual ligand responsiveness has been observed [17], [18]. Developmental profiling of zebrafish expression patterns [19] has also demonstrated a high degree of conservation between NR expression patterns in zebrafish and other vertebrate models.

The concepts underlying the screening technology described here were derived from previous studies conducted within the fruitfly, *Drosophila melanogaster*. In one of these studies [20], we showed it was possible to use fusions between the fly NR ligand binding domains (LBDs) and the DBD of Gal4 to visualize NR ligand and cofactor responsiveness in live animals using a Gal4-dependent GFP reporter. Two other studies, in which the NRs E75 [21] and DHNF4 [22] were affinity purified from insect cells or tissues, suggested that it might be possible to identify bound ligands using newly developed mass spectrometry techniques, provided that sufficient amounts and purities of the bound proteins could be achieved.

Here, we describe a unique combination of NR ligand sensor and affinity chromatography technologies within a vertebrate model. Using our TRβ and PPARγ lines, we show that they respond within different tissues to endogenous hormones and cofactors, as well as to exogenously added drugs. We also demonstrate the potential of our affinity purification system by isolating a transgenic receptor from zebrafish embryos and validating the function of one of the co-purified cofactors. We refer to our combination of technologies, within a pharmacologically receptive zebrafish model, as the “ligand trap” system.

## Results

### Assembly of the ‘ligand trap’ (LT) vector

**Figure 1:**
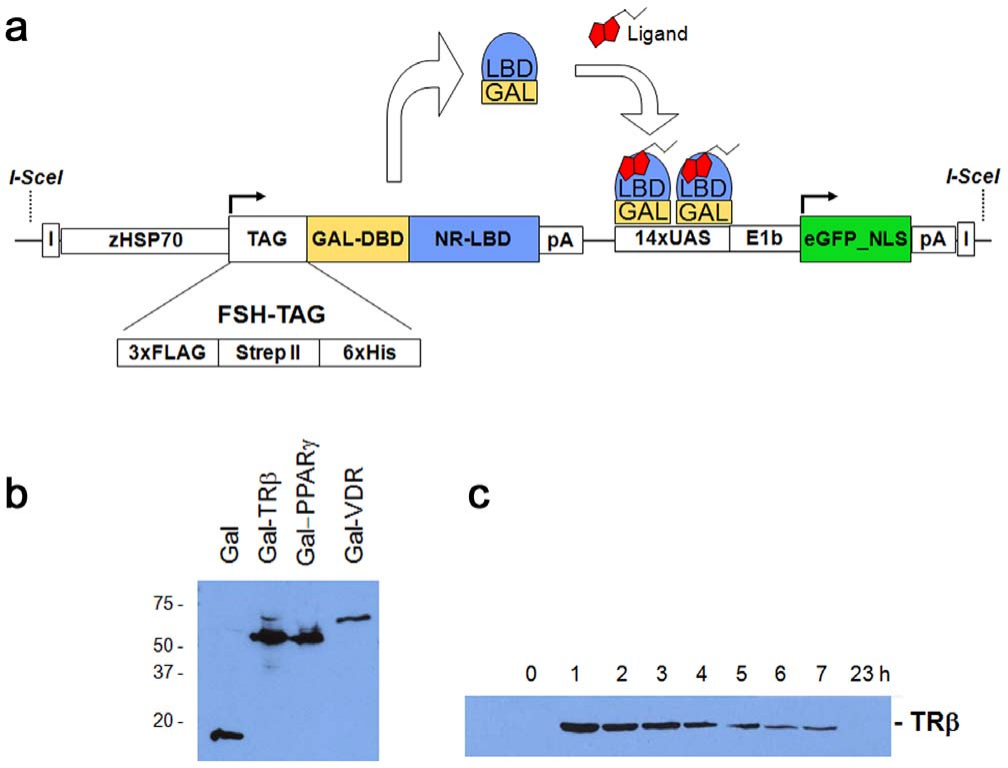
The Ligand Trap (LT) system. (**a**) Schematic diagram of the multi-component ligand trap (LT) construct. Upon heat pulse, the zebrafish *hsp70* promoter directs ubiquitous expression of the GAL4 DNA-binding domain (DBD) fused in-frame to a *human* nuclear receptor ligand-binding domain (LBD) and an affinity tag cassette (FSH-tag). Upon binding of this fusion protein to the GAL4 UAS (upstream activating sequence) response elements, in the presence of active hormone and cofactors, expression of the reporter gene (nuclear enhanced Green Fluorescent Protein (eGFP)) occurs. Expression of nuclear GFP is used to monitor receptor ligand sensor activity in a cell- and tissue autonomous manner in live zebrafish. The second component makes use of the tags to co-purify bound hormones or cofactors. I  =  insulator elements; pA  =  polyadenylation signal; NLS  =  nuclear localization signal. (**b**) Western blots of GAL4-NR fusion proteins. Embryos (F2; 72 hpf) were heat pulsed for 30 min at 37°C and recovered for 1 h at room temperature. 10 embryos were pooled and lysed in 50 µl of FSH buffer (see material and methods) followed by adding SDS buffer and boiling. Proteins were detected using the FLAG-M2 antibody. (**c**) Time course of fusion protein expression. TRβ embryos (F2; 72 hpf) were heat induced as in 1b) and recovered for the times indicated. Each sample contained 10 embryos.

Construction of the ligand trap (LT) vector (Figure 1a) involved an extensive series of sub-cloning events and configuration comparisons, with the chosen vector described below. The 3X FLAG [23], Strep II [24] and 6X His tags were combined into a single triple-tag cassette, collectively referred to as the ‘FSH’-tag, and inserted in front of a minimal GAL4 DNA-binding domain (DBD: residues 1–132). Following the Gal4 DBD sequence, a series of restriction sites, was used for the in-frame addition of NR cDNAs (hinge + LBD). To reduce potential lethality that might be caused by constitutive expression of the GAL4-LBD fusion proteins, a zebrafish *heat shock 70* gene promoter [25] was used for inducible expression in any tissue and at any developmental stage. Unique restriction sites flanking the *hsp70* promoter can be used to swap in tissue-specific promoters, if so desired.

Also within the vector is an enhanced GFP (eGFP; includes a nuclear localization signal) reporter gene that is expressed under the control of a 14x UAS_GAL4_-containing promoter. Expression of this reporter requires binding and activation of the Gal4-NR-LBD fusion protein. Bracketing the GAL-LBD fusion gene and the eGFP reporter are insulator elements that were introduced to discourage the influence of neighboring gene regulatory elements. Finally, *I-SceI* meganuclease recognition sites were added at the ends of the vector to facilitate integration into the genomes of transgene and meganuclease co-injected one-cell stage embryos [26].

### Induction of LT fusion protein expression

To test the heat inducible expression and activity of LT fusion proteins, and to determine the best parameters for activity screening, we generated stable transgenic lines for each human nuclear receptor by co-injecting the LT-vector together with *SceI* meganuclease into one-cell stage zebrafish embryos. Injected F0 fish were raised to adulthood and crossed with wild type fish to identify germline transformed animals. Positive progeny (F1) were identified either by target PCR or GFP screening (see Material and Methods). At least two independent lines for each nuclear receptor were obtained, unless otherwise noted. Transgenic LT embryos (F2) were heat pulsed at different developmental stages, temperatures and durations. Western blot detection using a M2-Flag antibody that recognizes the FSH-tag of each LT fusion protein revealed uniquely sized proteins with expected molecular weights (Figure 1b). Figure 1c shows the robust expression observed in homozygous transgenic (F2) Thyroid Receptor-β (TRβ; NR1A2) embryos at successive time points following a temperature shift to 37°C. As with most LT fusion proteins, peak expression was observed ∼1 hr following the heat pulse (Figure 1c). A thirty minute heat pulse at 37°C also proved adequate for the production of robust GFP fluorescence in the presence of endogenous or exogenously added ligands (see below).

### LT fusion proteins interact with fish ligands and cofactors

**Figure 2:**
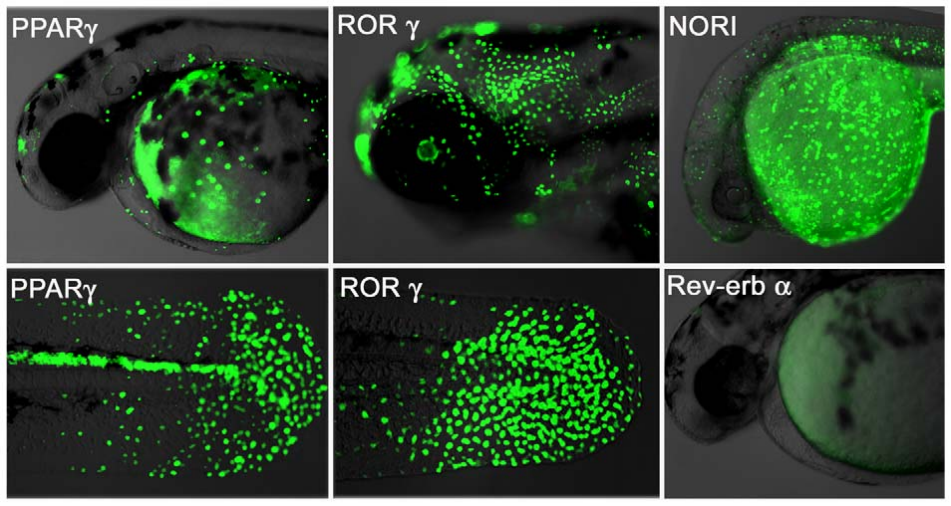
Human NRs interact with fish ligands and cofactors. Transgenic NR zebrafish show unique GFP patterns at different stages of development. Activity patterns of the PPARγ (first panel), RORγ (middle panel), NOR1 (upper right picture) and Rev-erbα (lower right picture) LT constructs. Embryos were heat pulsed at 37°C for 30 min and images taken 24 h later. PPARγ (48 hpf, F2) embryos show GFP expression in cells of the epidermis and heart, as well as in the posterior spinal cord. Strong GFP expression occurs in the epidermis and retina of RORγ embryos (72 hpf, F2). NOR1 embryos (F3) express GFP in the posterior spinal cord, hatching gland, epidermis and yolk syncictial layer (24 hpf, F2). Rev-erbα embryos show no GFP expression. Overlay pictures of bright field and GFP (75% transparent) are shown. Views are lateral with anterior to the left.

Different NR-derived lines should exhibit unique GFP responses during development in response to their respective ligand and cofactor distributions. To verify this, we induced expression of our Peroxisome Proliferator-Activated Receptor-gamma (PPARγ; NR1C3), Retinoic acid-related Orphan Receptor-gamma (RORγ: NR1F3), Neuron-derived Orphan Receptor-1 (NOR1: NR4A3) and Rev-erb alpha (Rev-erbα: NR1D1) transgenic lines and documented their patterns of GFP expression. The PPARγ embryos show strong GFP expression in the tail bud epidermis, as well as the brain, posterior spinal cord and heart (Figure 2; left panel). RORγ embryos show ubiquitous GFP expression over the entire embryonic epidermis during the first three days of development, along with brain and retina later on (Figure 2 middle panel). GFP expression in NOR1 embryos occurs in the epidermis and CNS (Figure 2; upper right picture). In contrast, Rev-erbα LT embryos show no GFP expression (Figure 2; lower right), consistent with its role as a transcriptional repressor [27].

To verify that GFP expression effectively reflects endogenous signaling activity, we injected a morpholino (MO) oligo-nucleotide, complementary to the translational start site of the transgene, into one-cell stage PPARγ, RORγ and TRβ embryos (Figure S1). GFP expression was lost or dramatically reduced in these MO injected embryos, even in the presence of control agonist.

Further documentation of these sites of hormone activity, and those of the other LT lines, should provide many new insights into novel NR functions and relationships during development. They will also serve as useful tools to genetically and chemically probe corresponding cellular and developmental processes, and to mark cells and tissues for co-expression and lineage analyses.

### Purification of an *in vivo* TRβ protein complex from early stage embryos

While a large number of nuclear receptor cofactors have been identified, their means of identification have generally been limited to immunoprecipitation from cultured cell extracts or yeast two-hybrid screens. The ability to identify cofactors throughout development or in different tissues where NRs are responding to unique cofactors, and/or ligands, would lead to the identification of many new cofactors, cofactor complexes and associated functions. To validate the usefulness of our tags, we induced expression of the TRβ fusion protein in 5–7 hpf embryos (Blastula/Gastrula stage) and purified the receptor protein complex from a whole embryo extract (see Methods for details).

**Figure 3:**
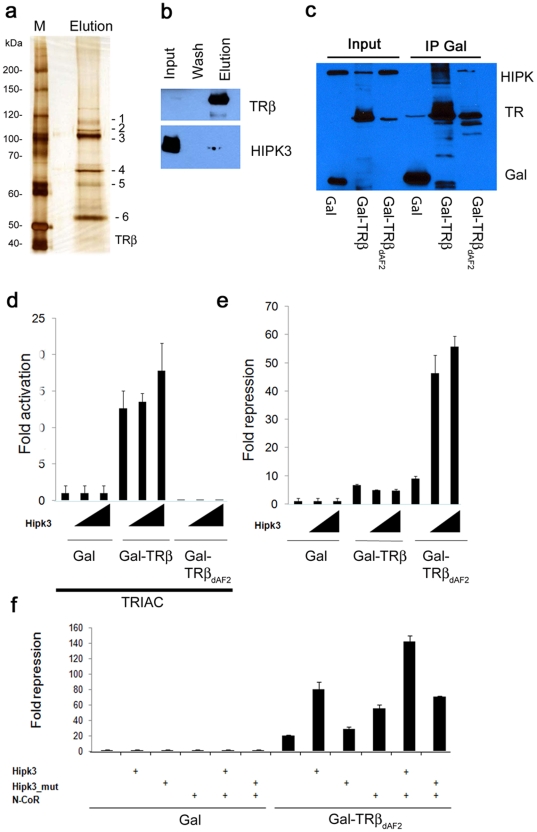
Co-purification of TRβ cofactors. (**a**) Affinity purification of TRβ from zebrafish embryos. Blastula-/Gastrula-stage embryos were heat induced for 20 min and recovered for 2 h at room temperature. After purification, the eluate was run on an 8% SDS PAGE and then silver stained. Protein bands (indicated as 1–6) were cut and in gel digested followed by mass spectrometry. Identified protein IDs are shown. M =  protein marker. (**b**) Immunodetection of HIPK3. Western blotting using a HIPK3 antibody (lower panel) shows that a subfraction of the protein co-purifies with the bait protein. Presence of the TRβ protein in the same fractions, detected using Flag-M2 antibody, is shown above. (**c**) HEK293 cells were transfected with FLAG-HIPK3 and one of three different Gal expression constructs containing the FSH-tag: Gal, Gal-TRβ (aa 189–461) and TRβ_ΔAF2_ (aa 189–451). After 48 h of transfection, the cells were harvested in IP buffer and Gal and Gal fusion proteins immunoprecipitated using Streptactin Sepharose. Western blotting using a FLAG M2 antibody shows HIPK3 in the Gal-TRβ and Gal-TRβ_ΔAF2_ pull downs. (**d**) Transcriptional activity of **T**Rβ proteins. 293 cells were transfected with a 2xUAS-Luciferase reporter construct (0.2 µg/well) and Gal or Gal-TRβ constructs (0.05 µg/well): Gal-TRβ (aa 189–461) and TRβ_ΔAF2_ (aa 189–451) in the presence of 100 nM TRIAC. Fold activation was measured in the presence (0.05 or 0.1 µg/well) or absence of HIPK3. Luciferase values were normalized against β-Gal. (**e**) Effects of HIPK3 on Gal-TRβ induced repression. 293 cells were transfected with a 2xUAS-Luciferase reporter construct and either Gal, Gal-TRβ or GAL-TRβ_ΔAF2_. Fold repression was measured in the presence or absence of HIPK3. Luciferase values were normalized against β-Gal. Transfections as under 3d. (**f**) The effect of HIPK3 was investigated in the absence or presence of the co-repressor N-CoR. Transfections were performed as in 3d using the Gal-TRβ_ΔAF2_ (aa 189–451) construct. Fold repression of the TRβ mutant in the presence of HIPK3 and/or N-CoR (0.05 µg/well) are shown.

Figure 3a shows the silver-stained SDS PAGE gel of the final elution of the TRβ purification. Several proteins (bands 1–5) in addition to the bait protein (band 6; Gal-hTRβ) can readily be seen. These and others were identified by MALDI-ToF and Orbitrap mass spectrometry (Dataset S1). Identified proteins included several known nuclear receptor interacting proteins, such as specific heat shock proteins and lung resistance protein [28], [29]. Nuclear proteins were in less abundance, due most likely to the use of whole cell extracts for this purification.

To test the validity and relevance of one of the novel TRβ cofactors identified, we focused on the protein Homeodomain-interacting Protein Kinase 3 (HIPK3). HIPK3 is a Ser/Thr kinase that affects transcriptional regulation, cell differentiation, growth and apoptosis [30], [31], [32], [33]. Notably, it has already been shown to bind and modulate two other NRs; Steroidogenic Factor-1 (SF1) and Androgen Receptor (AR) [30], [31].

Western blot analysis of the zebrafish extract and purified fractions (Figure 3b) confirms the presence of HIPK3 in the elution. Clearly though, the majority of HIPK3 present in the whole animal extract is not bound to the TRβ bait protein, consistent with its enzymatic participation in numerous other protein complexes.

The specificity and nature of this interaction was also tested using transiently expressed GAL, GAL-TRβ or GAL-TRβ_ΔAF2_ fusion proteins. The latter protein lacks the C-terminal AF2 helix, which is required for co-activator binding. The fusion proteins were expressed together with HIPK3 in human HEK 293 cells. Both full-length and AF2-deleted GAL-TRβ fusion proteins were able to pull down the HIPK3 protein (Figure 3c). Although the intensity of the HIPK3 band co-purified by the TRβ_ΔAF2_ deleted protein appears less intense, this is largely due to the presence of additional co-migrating bands in the full-length TRβ pull down, which may be due to subsequent modifications of the non-deleted LBD protein. We conclude that the AF-2 helix is not required for HIPK3 complex formation.

### HipK3 negatively modulates TRβ transcriptional activity

Ligand activation of TR is associated with the displacement of co-repressors and recruitment of co-activators. To investigate whether HIPK3 affects the transcriptional activity of TRβ, a cell culture co-transfection assay was employed using Gal4-TRβ and Gal-TRβ_ΔAF-2_ fusion protein. Deletion of the AF2 domain of TRβ results in continued co-repressor binding even in the presence of hormone, a phenomenon referred to as ‘Resistance to Thyroid Hormone’ (RTH) [34]. This type of deletion represents the most severe genetic form of RTH.

Addition of the thyroid hormone analog, TRIAC (3,5,3′-triiodothyroacetic acid) strongly stimulates the activity of the full LBD fusion protein, but has no effect on the non-activating AF2-deleted form (Figure 3d). Addition of HIPK3 led to a modest (∼1.3X) increase in the transcriptional activation activity of the WT fusion protein (Figure 3d). No effect was seen upon addition of HIPK3 on the activation activity of the AF2 deleted protein, consistent with the need for the AF2 motif for agonist-based transcriptional activation.

As TRβ represses the expression of target genes in the absence of hormone, we also looked for effects of HIPK3 on Gal4-LBD fusion protein activity using a reporter plasmid with significant basal transcription activity, making it suitable for the observation of repression (Figure 3e and Figure S2a). As with the transcriptional activation assay with ligand present, HIPK3 had only a modest affect on GAL4-TRβ activity (∼1.3X lower). However, with the AF2 removed, HIPK3 increased transcriptional repression by an impressive 5–6 fold. This suggests that HIPK3 acts primarily as an enhancer of TRβ- mediated transcriptional repression.

To further probe the mechanistic nature of this HIPK3 affect, we examined its ability to augment repression mediated by the known TRβ co-repressor, N-CoR [35], [36], [37]. Co-expression of HIPK3 or N-CoR with the WT TRβ fusion protein increased repression of the UAS-containing reporter approximately 4- and 3- fold respectively (Figure 3f and Figure S2b). When added at the same time, a further ∼2-fold increase in repression mediated by the TRβ_ΔAF2_ mutant was observed. These interactions and effects were not observed with a kinase-defective HIPK3 protein (Figure 3f). These results are consistent with the activities of other HIPK family members on target gene expression and co-repressor function [38], [39].

### Zebrafish ligand trap activities are hormone-inducible

**Figure 4:**
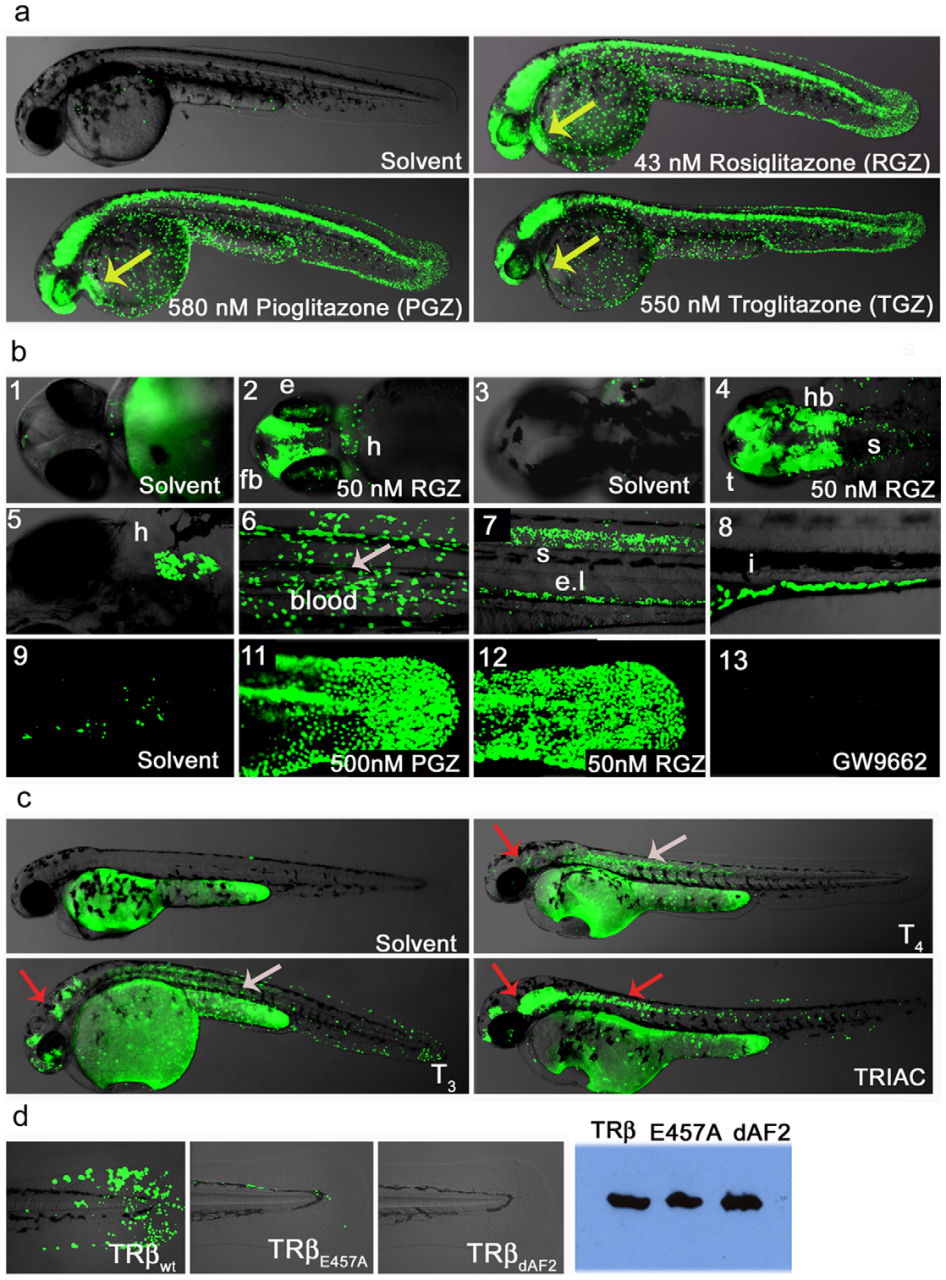
Ligand trap activities are drug responsive and reveal SNRM activities. (**a**) LT-PPARγ embryos show strong reporter activation in the presence of receptor specific agonist (uses as indicated). After heat pulse followed by overnight incubation with solvent or drug GFP expression in cells of the epidermis, CNS, eye, blood and heart (48 hpf; lateral view) is induced. Yellow arrow indicates GFP expression in heart. (**b**) Upper panel shows magnifications of embryo heads in ventral (b-1-2) and dorsal (b-3-4) orientation of 2 dpf fish treated with solvent or 50 nM Rosiglitazone (RGZ). B-2 shows reporter expression in eye (e), forebrain (fb), heart (h) and b-4 indicates GFP expression in hindbrain (hb), tectum (t) and anterior spinal cord (s). Middle panel shows drug responses in tissues of older embryos: Expression in heart (b-5; 5 dpf ventral view), in blood (b-6; 8 dpf), in the spinal cord (s) and endothelium layer ((el), b-7; 4 dpf, lateral view) and in the intestine ((I, b-8; 6 dpf). Embryos were subjected to a 30 minute heat pulse at 37°C, followed by incubation with 50 nM Rosiglitazone for 24 h. The lower panels show responses to: endogenous hormone in the tail epidermis of 3 dpf embryos treated with solvent (b-9); 500 nM Pioglitazone (b-11), 500 nM RGZ (b-12) and treatment with the antagonist GW9662 (500 nM). (**c**) TRβ LT embryos show tissue-specific responses to hormones and drugs. After 40 min heat induction, embryos (24 hpf) were incubated with compounds for 28 h in a 28°C incubator in the dark. Solvent (Ethanol) treated embryos show no GFP expression. T_4_ (2.5 µM) treated embryos show strong reporter activity in muscle and to a lesser amount in epidermis, brain and eye retina. T_3_ (2.5 µM) treatments result in similar responses but GFP induction in epidermis, brain and eye is stronger, and signal is also seen in blood cells. TRIAC (100 nM) treatments also induce GFP responses in brain, heart, eye, epidermis and muscle, and in addition, anterior spinal cord. Overlay pictures of bright field and GFP (75% transparent) are shown. Arrows indicate tissue-specific GFP responses: grey  =  muscle and red  =  brain. (**d**) TRβ responses in transgenic fish are dependent on a functional LBD. TRβ_wt_, TRβ_E457A_ and TRβ_dAF2_ embryos (24 hfp, F1) were heat pulsed and soaked in TRIAC (100 nM) as described in Figure 4c. The upper row shows strong (wt), weak (E457A) and no (dAF2) epidermal GFP expression in the tail. Western blot detected proteins show similar levels of TRβ transgene expression (right side).

To test whether our LT lines are responsive to externally provided hormones or drugs, we treated the PPARγ line with readily available agonists and antagonists (Figure 4a–b). PPARγ fish treated with the receptor specific agonists Rosiglitazone (RGZ), Pioglitazone (PGZ) or Troglitazone (TGZ)) increased the GFP reporter response from the relatively restricted signal in tail epidermis and posterior spine to include strong expression in the CNS, heart, blood, renal tube and eye (Figure 4a). Higher magnification of the embryo head shows specific and detailed activation in the presence of drug in the brain, eye and heart (Figure 4b panels 2 and 4). Older embryos (5–6 dpf) also show strong GFP expression in other tissues including heart, blood and intestine (Figure 4b, panels 5–8). Treatment of PPARγ fish with PPARα- or PPARδ-specific agonists showed no increase in reporter activation when used at their receptor-specific EC_50_ concentrations (Figure S3a). However, as seen in Gal4-PPAR cell based assays [40], higher concentrations of the beta or alpha agonist do result in partial activation of the PPARγ LT embryos (Figure S3a). Treatment with the selective PPARγ antagonist GW9662 either decreases or completely blocks the GFP responses to endogenous ligand(s) (Figure 4b-13 and Figure S3b). When added together with Rosiglitazone, in a classical agonist/antagonist competition experiment, GW9662 was clearly able to displace the potent PPARγ agonist (Figure S3b).

As further validation of drug responsiveness, we also treated TRβ fish with known agonists (Figure 4c). Vehicle treated TRβ embryos show the same response seen in untreated embryos. TRβ fish treated with either Thyroxine (T_4_) or Triiodothyronine (T_3_) induced reporter activation in the eye, heart, epidermis, blood, muscle and brain (Figure 4c). As expected, T_3_ activates the reporter more strongly than T_4_. Treatments with TRIAC, also yielded strong responses at dosages similar to those used in mammalian tissues. The eye activity of TRβ ligand trap fish correlates well with previously reported expression of endogenous TRβ in retina at 48 hpf [19]. However, our finding of receptor activity in other tissues such as the brain and anterior spinal cord are novel. Interestingly, in a recent profiling of zebrafish NR expression patterns, Bertrand and colleagues found that nuclear receptors expressed in the retina, except of TRβ, are also expressed in the brain and/or anterior spinal cord. We expect that many of the novel activity patterns observed in our LT lines will represent novel roles in early development, opening the door for new avenues of study.

### LT drug treatments reveal tissue selective nuclear receptor activities

Recent drug discovery programs have been heavily focused on the identification of compounds with activities in a subset NR-regulated tissues. Such **S**elective **NR** **M**odulators (SNRMs) would yield fewer side effects and have additional uses. Interestingly, each of the TR agonists tested above exhibited significant tissue-selective activities, as indicated by the colored arrows in Figure 4c. For example, T_3_ and T_4_ elicited strong GFP responses in dorsal muscle while TRIAC did not. Conversely, TRIAC elicited responses in the spinal cord while T_3_ and T_4_ did not. Differential responses were also seen in the eye, heart, epidermis brain and blood. Many of these sites of action have not been previously documented.

To further verify that GFP expression in the TRβ lines are ligand- and cofactor-dependent, we made two additional transgenic lines carrying mutations in the TRβ LBD (Figure 4d). These include the previously described LT-TRβ_ΔAF2_ and a construct carrying a single AF2 point mutation, LT-TRβ_E457A_. Both mutations result in impaired co-activator recruitment upon ligand binding [34]. As already shown (Figure 4c), TRβ WT embryos respond strongly to the addition of TRIAC. However, TRIAC-treated TRβ_E457A_ fish show dramatically reduced GFP expression, with signal restricted mainly to epidermal cells of the tail bud, and TRβ_ΔAF2_ embryos show no detectable GFP whatsoever (Figure 4d). Western blot analysis using anti-FLAG antibody shows that both fusion proteins are well expressed. We conclude that the signals observed with these lines are ligand and co-activator dependent.

### GFP signals are readily quantified and suitable for drug screening

**Figure 5:**
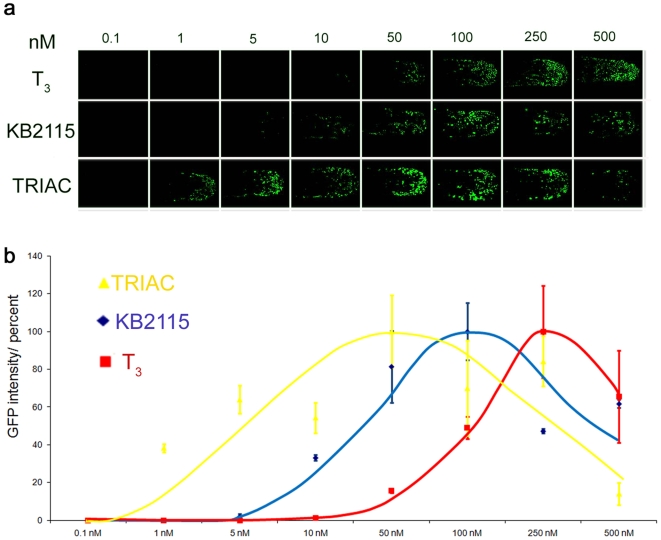
LT drug responses are quantifiable. (**a**) Dose dependent GFP expression Shown are the dose responses for T_3_, KB2115 and TRIAC in TRβ tail epidermis (48 hpf) treated embryos. Treatments were done from 0.1–500 nM. GFP images show anterior-posterior tail view (imaged at 150× magnification) (**b**) The graph indicates average intensity of GFP signals for each treatment in percent. GFP expression was quantified using nuclei count software from MetaX files. Values for drug treated fish are shown in yellow (TRIAC), in blue (KB2115) and in red (T_3_).

To test whether drug effects can be quantified with our ligand trap fish, we subjected TRβ LT embryos to increasing concentrations of the agonists T_3_, TRIAC or KB2115 [41], and the antagonist Amiodarone. Figure 5a shows a series of tail regions of the treated TRβ embryos. Note that the number and intensity of GFP responding nuclei increase in proportion to hormone concentration but decrease again as levels become teratogenic.

Figure 5b shows averaged GFP response curves for increasing concentrations of all three ligands. TRIAC proved to be the most effective, then KB2115, and lastly T_3_. Amiodarone treatment yielded no detectable GFP response as expected. Importantly our dose-response curves for the various TRβ agonists tested correlate well with previously determined animal and human data [42], [43].

## Discussion

**Figure 6:**
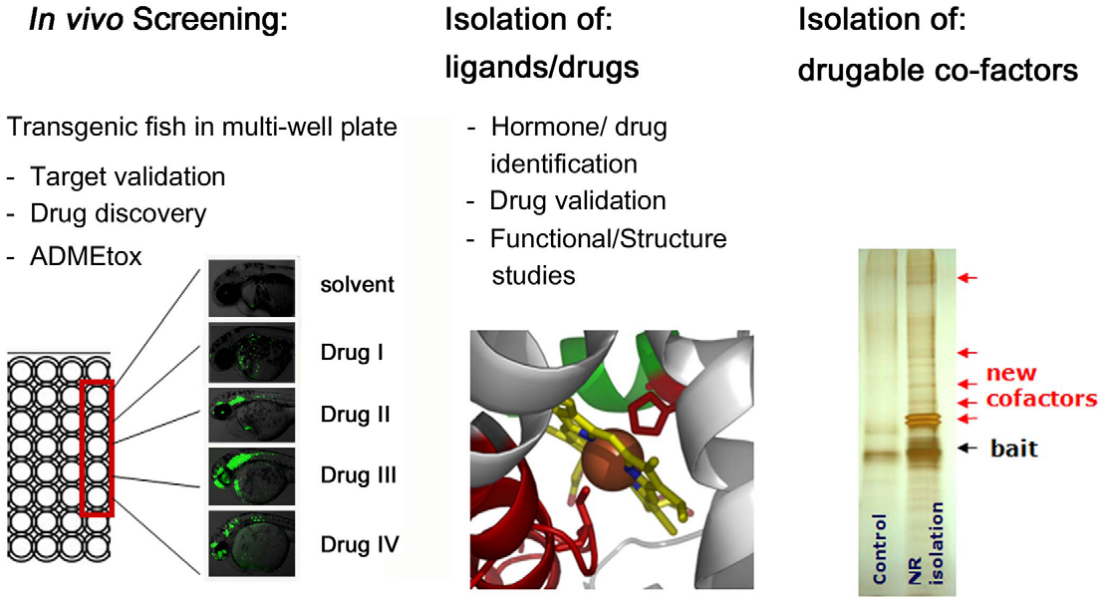
Schematic representation of LT platform functions.

We have taken advantage of the unique properties of zebrafish as a model organism to engineer a powerful *in vivo* screening system for new Nuclear Receptor ligands and cofactors (uses summarized in Figure 6). Embryos and larvae produced by the transgenic fish are readily arrayed, drugged and assayed within microtiter plates, making them suitable for high throughput screens. As discussed below, this whole-animal approach will facilitate the identification of ligands and cofactors that could not otherwise be identified by previously used *in vitro* or cell based approaches. Many of these ligands and cofactors will act tissue- or stage-specifically, providing new options for NR study and manipulation. In turn, the tissue-specific GFP responses produced by these novel endogenous and exogenous ligands will also provide the bases for subsequent genetic, molecular and visual screens that provide new insights into how these tissues develop and function.

A major problem with current drugs that target NRs is the side-effects that arise due to unwanted modulation of the targeted NR in tissues that are not the source of the problem, or to the targeting of other NR members of the same class. The identification of more selective compounds that act more NR- and tissue-specifically has the potential to circumvent these problems. Although such selective NR modulators (SNRMs) have been discovered, they are extremely difficult to screen for and new techniques and models are needed to succeed in the discovery of beneficial new drugs. With the LT system, known or potential ligands can be tested in all tissues and during any stage of development. As shown here, this readily reveals the SNRM properties of all active compounds.

The LT lines developed here should also be immensely useful for the identification of ‘endocrine disruptors’ within industrial, agricultural and municipal wastes [44]. The majority of these molecules emulate NR ligands, thereby interfering with normal human and animal development and function. Fish are one of the major targets and concentrators of endocrine disrupting compounds, and as such, are a highly appropriate test bed for their presence [45].

Several methods for isolating native protein complexes have been described in yeast, bacteria, cell culture, plants and insects [46]. Here, we show that the affinity tag cassette used in the LT system is capable of purifying protein complexes from whole vertebrate tissues with sufficient yield and purity for subsequent cofactor purification and identification. With appropriate modifications, the identities of bound ligands should also be readily determined. Indeed, the use of multiple affinity chromatography steps followed by mass spectrometry has already yielded ligands for the *Drosophila* NR E75 [21], and the mouse NR PPARα [47]. Given the number of NRs that remain as orphans, this has great potential for the timely identification of their recalcitrant ligands. The ability to identify directly bound ligands also provides a means to determine whether active compounds identified in compound screens are directly bound and unmodified. In some cases, the active molecule may be a metabolite of the original compound added. Thus, the LT system has the potential to identify ‘pro-drugs’, something that is difficult or impossible to achieve by other means.

Our results with TRβ suggest that its major role during early development is primarily as a repressor. This was evidenced by the lack of GFP activity in the absence of supplemented agonists, and the isolation of repressive cofactors. Among the novel cofactors identified was the kinase HIPK3, which we found to act as a potentiator of target gene repression. Given the ligand-independent nature of this interaction, it may provide additional means to treat or circumvent ‘resistance to thyroid hormone’ (RTH) conditions. Previous studies on HIPK proteins have also shown important functions during development [48], and in some cases via interactions with well characterized co-repressors [38]. As the dissociation and disposal of these co-repressors appears to require phosphorylation and/or ubiquitination [49], [50], [51], HIPK3 may be functioning in a related capacity. It should be noted, however, that while HIPK3 enhances N-CoR-mediated repression of TRβ, it has also been shown to mediate ligand dependent transcriptional activation by the Androgen and SF1 Nuclear Receptors, while having no apparent effect on Glucocoritcoid Receptor mediated transcription [30], [31]. As such, NR modulation by HIP kinases may serve as an alternative means of differentially controlling multiple NR activities.

Taken together, this study shows that the LT system provides enormous potential for the acceleration of NR functional analyses. Its amenability to systems biology scale analyses that access all tissues and developmental processes will reveal many new unpredictable and unexpected results.

## Materials and Methods

### pLT plasmid construction

To generate the ligand trap vector, p-LT, we first combined the SV40 late polyadenlyation site from pCS2+ (cloned XbaI/Hind III) and the Gal4 DBD (aa 1–132; cloned into XbaI) into pBluescript (Stratagene). PmeI and NheI restriction sites were introduced in the primer as cloning sites for NR ligand binding domains. An EcoRV restriction site was designed N-terminal to the Gal4DBD for introducing the FSH-triple tag. Oligos for the triple tag were designed with EcoRI restriction sites on both ends and annealed together followed cloning into EcoRI digested pBSII. The tag was then PCR amplified with primers containing EcoRV restriction sites and a Kozak consensus sequence followed by an ATG start codon. This fragment was then introduced into the EcoRV restriction site of pBSGal1-132-pA, resulting in pBS-tripletag-Gal1-132-pA. Next, two *I-SceI* mega nuclease recognition sites were introduced by site directed mutagenesis flanking the fragment.

The eGFP-NLS gene was PCR amplified from the pUAS Stinger vector [52] and cloned into pBluescript II containing a SV40 polyA to generate pB-eGFP-pA. A fragment containing multi-UAS Gal DBD binding sites followed by the basal adenovirus promoter, E1b, was PCR amplified from pBUASEIB [13] and inserted into pB-eGFP-pA. The UASE1b-eGFP-pA fragment was then cut out by KpnI digestion and cloned into KpnI digested pB *SceI*-Tag-Gal1-132-pA. The resulting plasmid was SacII/NotI cut to insert a SacII/NotI fragment of the zebrafish Hsp70 promoter amplified from pzHSP70/4prom [25]. Finally, the gypsy insulator elements from the UAS-Stinger GFP transformation vector were PCR amplified and inserted upstream of the Hsp70 promoter into SacII restriction sites or downstream of the eGFP reporter into HindIII/ApaI.

### Fish microinjections and maintenance

Zebrafish were maintained at 28.5°C on a 14/10 hour light/dark cycle and staged according to hours (h) or days (d) postfertilization [53]. To generate pLT lines, plasmids were pre-digested with *I-SceI* (0.8 µg DNA, 1 µl *I-SceI* (New England Biolabs) 2 µl 10x *I-SceI* buffer, 1 µl BSA) for 20 min at 37°C. Digested DNA was adjusted with 0.1% phenol red. 4.6 nl of DNA solution was injected into the blastomeres of early one-cell stage embryos [26]. Morpholino (MO) injection: 4.6 nl of MO (0.5 mM) in 0.1% phenol red was injected into the one-cell stage.

F0 fish were crossed with WT fish to identify germ-line transformed animals, as determined by fin cut PCR with GFP primers or control agonist treatments. F1 progeny showing strong and consistent GFP responses were selected for F2 homozygote production. To avoid reporter GFP silencing of stable transgenic LT lines [54], [55], F2 lines showing strong and consistent GFP responses were selected for continued propagation of the line. These lines have been maintained for several generations with no loss of responsiveness. At least two independent stable lines of each LT construct were generated, however for LT-PPARγ only one germline fish was identified.

All experiments were approved by the institutional review board at University of Toronto.

### Plasmids, cell culture transfection

FSH-Gal1-132 from pLT plasmid (NotI/PmeI) was cloned in frame into pcDNA3.1 (Invitrogen) to generate pcDNA3 FSH-GalDBD. The N-CoR plasmid has been described previously [36]. pFLAG-HIPK3 (2–1191) and pFLAG-HIPK3 (K226R) were provided by J.J. Palvimo (University of Helsinki). The 14xUASE1B plasmid was a gift of Dr. Sue (Banting and Best Department of Medical Research/originally provided by Dr. R.W. Köster; Helmholtz Zentrum, Germany). The plasmids for reporter gene assays 2X-GAL4 binding-site luciferase, pSV40 β-Gal) have been described previously [37], [56]. 293 cell line transfections were done with Lipofectamin™ 2000 (Invitrogen).

### Compound screening

Embryos were heat induced (28→37°C) for 30 minutes and then arrayed in 24 well plates (10 per well). The water was removed and 500 µl of embryo water/well including dissolved small molecules or solvent was added shortly after heat pulse. Embryos were incubated at 28°C for 14–20 h and then monitored using a fluorescent dissection scope (SteREO Lumar.V12 Carl Zeiss). For analyzing GFP fluorescent pattern, embryos, larvae and adult fish were anesthetized with Tricaine (Sigma, Cat.# A-5040). Embryos were mounted in 1% AgarPlaque PlusTM Agarose (# 21403A) purchased from PharminGen.

### Drugs used

Sigma-Aldrich: Amiodarone hydrochloride (A8423); T_3_ (T2877); T_4_ (T2376) and TRIAC (T7650); Alexis Biochemicals: Pioglitazone (ALX-270-367); Cayman Chemicals: Rosiglitazone (#71740); Troglitazone (#71750); GW 9662 (#70785); KB2115 (#10011054), GW 0742 (#10006798) and GW 590735 (#10009880).

### Affinity purification of proteins

Purifications were performed at 4°C. 5-6 hpf LT-TRβ embryos (128 g) were heat induced (37°C) for 20 min and recovered for 2 h. Homogenization was carried out in 100 ml FSH buffer (100 mM Tris-Cl pH 8, 150 mM NaCl, 0.1% Triton X-100 and Roche complete Mine protease inhibitor cocktail) including 2.5 mM DTT, 10 nM Avidin and 1 mM EDTA. After 20 min incubation the lysate was centrifuged for 10 min at 9000 rpm. 15 ml of prewashed (FSH buffer) Agarose-bead slurry (ABT; 2% plain Agarose beads) was incubated with the centrifugation supernatant (220 ml) for 30 minutes. After this incubation and again centrifugation the supernatant (200 ml) was incubated with 5 ml prewashed Strep Tactin® (IBA; Cat: 2-1201-0251) slurry for 2 h. After incubation the beads were transferred to 2 gravity disposable columns (Bio-Spin columns, Cat: 732–6008) and washed 6 times with 2 ml of FSH-buffer including 1 mM EDTA. Bound proteins were eluted using volume 0.5 ml of 2.5 mM d-desthiobiotin containing FSH-buffer and 1 mM EDTA. For triple step purifications the Strep elutions were incubated for 2 hrs with FLAG M2 monoclonal antibody matrix (Sigma), and then washed 3times with FSH buffer followed by elution with FSH buffer containing 300 µg/ml 3x FLAG peptide (Sigma). Flag elutions were incubated for 1 hrs with 60 µl of Talon beads in an Eppendorf tube, and then washed two times with FSH buffer followed elution with 2xSDS buffer. For immunoprecipitations, HEK 293 cells were grown in 10 cm dishes and DNA (9 µg of FLAG-HIPK3 with either 4 µg of pcDNA3-FSH-GAL, -GAL-TRβ or -GAL-TRβ_ΔAF2_/empty vector was used to adjust the DNA concentration) was transfected with Lipofectamin following the manufacturer's instructions. Cells were harvested in 1 ml IP buffer (50 mM Tris pH 8, 150 mM NaCl, 1 mM EDTA, 0,75% NP-40, 1 mM DTT and Roche complete Mine protease inhibitor cocktail). After 10 min centrifugation at 13,000 rpm, 600 µl of cleared extract was incubated with 100 µl Strep Tactin® slurry for 2 h; followed by 3 washes with 1 ml of IP buffer. Immunoprecipitated proteins were separated by SDS PAGE and detected by FLAG-M2 Western blotting.

### Western blotting

Expression of ligand trap constructs was verified using a monoclonal mouse αM2 antibody (dilution 1∶10 000; Sigma). Heat pulsed and recovered transgenic embryos were homogenized in 5 µl FSH buffer/embryo and denatured with SDS PAGE loading buffer followed separation on 10% SDS-polyacrylamide gels. Detection of HIPK3 was done with αHIPK3 from Abgent (AP 7500b).

### Mass spectrometry

Eluted proteins were separated by SDS PAGE, silver stained and individual bands excised, digested with Trypsin and analyzed by MALD-TOF or ESI-MS tandem mass spectrometry. All samples for ESI-MS were analyzed by a 2-hour LC gradient using a split-free nano-LC (EasyLC, Proxeon, Odense, Denmark) coupled to a LTQ-Orbitrap XL, as recently described [57]. Raw data was converted to m/z XML using ReAdW and searched on a Swquest SorcererTM, using both Sequest and X!Tandem, against a zebrafish IPI protein sequence database (version 3.38) also containing the protein sequences of known contaminants (i.e. human keratins) and trypsin and the bait protein (human thyroid receptor). Searches were performed with a fragment ion mass tolerance of 0.8 Da and a parent ion tolerance of 50 ppm. Complete tryptic digestion was assumed. The iodoacetamide derivative of cysteine was specified in Sequest and X!Tandem as a fixed modification. The oxidation of methionine was specified as a variable modification. Scaffold (version Scaffold 2.1.1, Proteome Software Inc., Portland, OR) was used to validate MS/MS based peptide and protein identifications. Peptide identifications were accepted if they could be established at greater than 95.0% probability as specified by the Peptide Prophet algorithm and contained at least 1 identified peptide. Proteins that contained similar peptides and could not be differentiated based on MS/MS analysis alone were grouped to satisfy the principles of parsimony.

### Oligos used to clone pLT:

pB Gal1-132-pA:

5’ATTCATCTAGAGATATCAAGCTACTGTCTTCTATCGAACAAGC 3’ATTATCTAGAGTTTAAACAGCTAGCTGATGATGTCGCACTTATTCTATGC

pB II triple tag:

sense –AATTCGACTACAAAGACCATGACGGTGATTATAAAGATCATGACATCG -

ACTACAAGGATGACGATGACAAGGAGAACCTGTACTTCCAGTCCAACTGGAGCC -

ACCCGCAGTTCGAAAAGCATCACCATCACCATCACG

-antisense -ATGATCTTTATAATCACCGTCATGGTCTTTGTAGTCG

-AGTTGGACTGGAAGTACAGGTTCTCCTTGTCATCGTCATCCTTGTAGTCGATGTC

-AATTCGTGATGGTGATGGTGATGCTTTTCGAACTGCGGGTGGCTCC

pB triple tag-Gal1-132-pA

5’ ATTATGATATCgccaccatgGACTACAAAGACCATGACGG

3’ ATTATGATATCGTGATGGTGATGGTGATGC

SceI pB triple tag-Gal1-132-pA

T7 5’ GACTCACTATAGGGCTAGGGATAACAGGGTAATGAATTGGGTACCGGG-

T7 3’ CCCGGTACCCAATTCATTACCCTGTTATCCCTAGCCCTATAGTGAGTC-

T3 5’ CGGTGGAGCTCCAGTAGGGATAACAGGGTAATCTTTTGTTCCCTTTAGTG-

T3 3’ CACTAAAGGGAACAAAAGATTACCCTGTTATCCCTACTGGAGCTCCACCG-

pB eGFP-pA

5' ATTATCTAGAACCATGGTGAGCAAGGGC

3' ATTATCTAGATTACTTGTACAAGTAGCG

pB UASE1b-eGFP-pA

5' ATTATCCGCGGGGTACCCTCCAAGGCGGAGTACTGTCC

3’ ATAATCGGCCGGTGTGGAGGAGCTCAAAGTGAGGC

pLT ; SceI pB zHsp70 triple tag Gal-1-132-pA-UASE1b-eGFP-pA)

5’ATTATCCGCGGTCAGGGGTGTCGCTTGG 3’ATTATGCGGCCGCGATATCGAATTCCTGCAGG

pLT gypsy; SceI gypsy pB zHsp70 triple tag Gal-1-132-pA-UASE1b-eGFP-pA)

5’ Hsp ATAACCGCGGTCACGTAATAAGTGTGCG

3’ Hsp ATAACCGCGGAGATCTATACTAGAATTGATCGGC

5’ Gfp ATAAAAGCTT TCACGTAATAAGTGTGCG

3’ Gfp ATAAGGGCCCATACTAGAATTGATCGGC

Morpholino oligo sequence:

5' to 3' and complementary to the translational start of the FSH Gal-NR:

ACCGTCATGGTCTTTGTAGTCCATG

Primes used to clone NR cDNAs:

NR1A2_wt_/ _E457A_/_ dAF2_

5’ ATTAGCTAGCATGACTCCCAACAGTATGAC

NR1A2wt

3’ ATTAGTTTAAACCTAATCCTCGAACACTTCC

NR1A2_E457A_

3’ ATTAGTTTAAACCTAATCCTCGAACACTGCCAAGAACAAAGG

NR1A2_dAF2_

3’ ATTAGTTTAAACCTACTATTCTGTGGGGCATTCCACC

NR1C3

5’ ATTAGCTAGCGAGAAGGAGAAGCTGTTGG

3’ ATTAGTTTAAACCTAGTACAAGTCCTTGTAGATCTCC

NR1F3

5’ ATTAGCTAGCATGTCCAAGAAGCAGAGGG

3’ ATTAGTTTAAACTCACTTGGACAGCCCCACAGG

NR1D1

5’ ATTAGCTAGCATGCTTGCTGAGATGCAGAGTGCC

3’ ATTAGTTTAAACTCACTGGGCGTCCACCCGGAAGG

NR4A3

5’ GATCGCTAGCCCATTACAACAGGAACCTTCTCAG

3’ CACCGTTTAAACTTAGAAAGGTAGGGTGTCCAGG

## Supporting Information

Figure S1 Ligand trap signals are NRs specific A Morpholino (MO) against the FSH-tag of the GAL-NR transgene was injected into one-cell stage F3 embryos of LT-PPARγ, RORγ and LT-TRβ. Endogenous and agonistic drug responses were compared between control and MO injected embryos. The upper row shows 48 hpf PPARγ embryos in their chorions, and tail close ups of the same conditions are shown in the middle row. The lower row shows RORγ (F2) or homozygous TRβ F3 embryos in the presence of 250 nM T3 with control or MO injections.
(0.70 MB TIF)

Figure S2 (a/b) Expression of HIPK3 and Gal- and Gal-TR-fusion proteins in reporter assays (Figure 3e and 3f) verified by Western Blot using Flag M2 antibody.
(1.62 MB TIF)

Figure S3 Selective PPARγ drug responses (a) At 24 hpf PPARγ embryos were subjected to a 30 minute heat shock at 37°C and then incubated for 24 hr in the presence of agonists specific for one of the three PPAR isoforms (Rosiglitazone for γ, GW0742 for δ/β and GW9578 or GW590735 for α). The concentrations chosen represent known EC50 values for the appropriate targets, along with significantly higher levels to test for cross-reactivity. Lateral views of 48 hpf embryos, anterior to the left, are shown. (b) PPARγ agonist/antagonist replacement. At 24 hpf PPARγ embryos were subjected to a 30 minute heat induction at 37°C and either incubated for 24 hr in the presence of solvent or 1000 nM GW9662 (upper row) or 50 nM Rosiglitazone (RGZ) alone or 50 nM RGZ and increasing concentrations of GW9662 (20 nM and 500 nM). Two embryos for each treatment showing lateral views at 48 hpf, anterior to the left, are shown.
(2.76 MB TIF)

Dataset S1 Table of mass spectrometry-identified bait protein (Gal-hTRβ) and interacting proteins are shown. Eluted proteins were separated by SDS PAGE, silver stained and individual bands (band 1–6) excised, digested with Trypsin and analyzed by MALD-TOF or ESI-MS tandem mass spectrometry. Accession number, molecular weight (MW) and description of identified proteins are listed. The purified bait (Gal-hTRβ) and the investigated cofactor are underlined in yellow.
(0.04 MB XLS)
