# Supplementary figures and images for: A Live Zebrafish-Based Screening System for Human Nuclear Receptor Ligand and Cofactor Discovery
Source: PLoS One. 2010 Mar 22;5(3):e9797. doi: 10.1371/journal.pone.0009797 (PMC2842432; doi:10.1371/journal.pone.0009797)

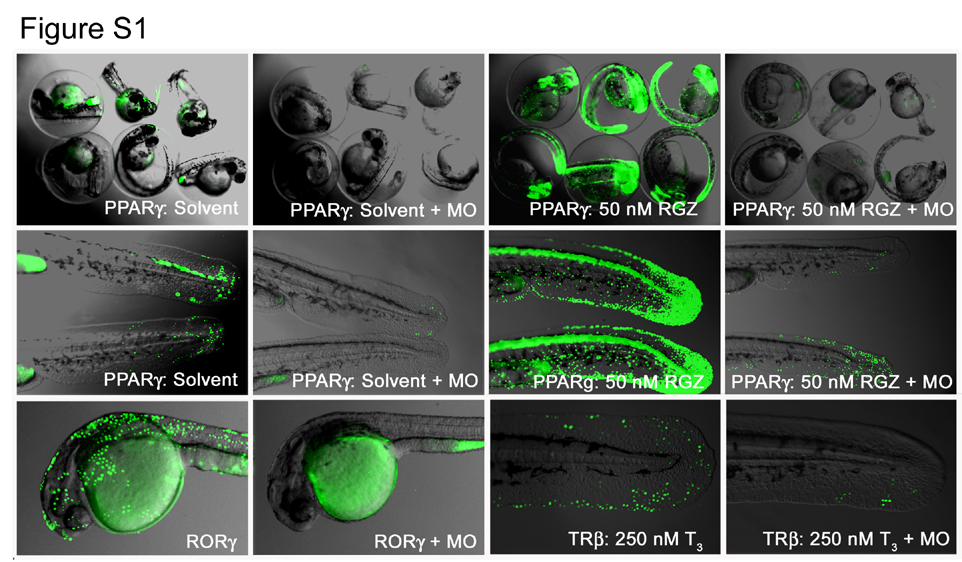

Supplement: Figure S1 — Ligand trap signals are NRs specific A Morpholino (MO) against the FSH-tag of the GAL-NR transgene was injected into one-cell stage F3 embryos of LT-PPARγ, RORγ and LT-TRβ. Endogenous and agonistic drug responses were compared between control and MO injected embryos. The upper row shows 48 hpf PPARγ embryos in their chorions, and tail close ups of the same conditions are shown in the middle row. The lower row shows RORγ (F2) or homozygous TRβ F3 embryos in the presence of 250 nM T3 with control or MO injections. (0.70 MB TIF) [file pone.0009797.s001.tif]

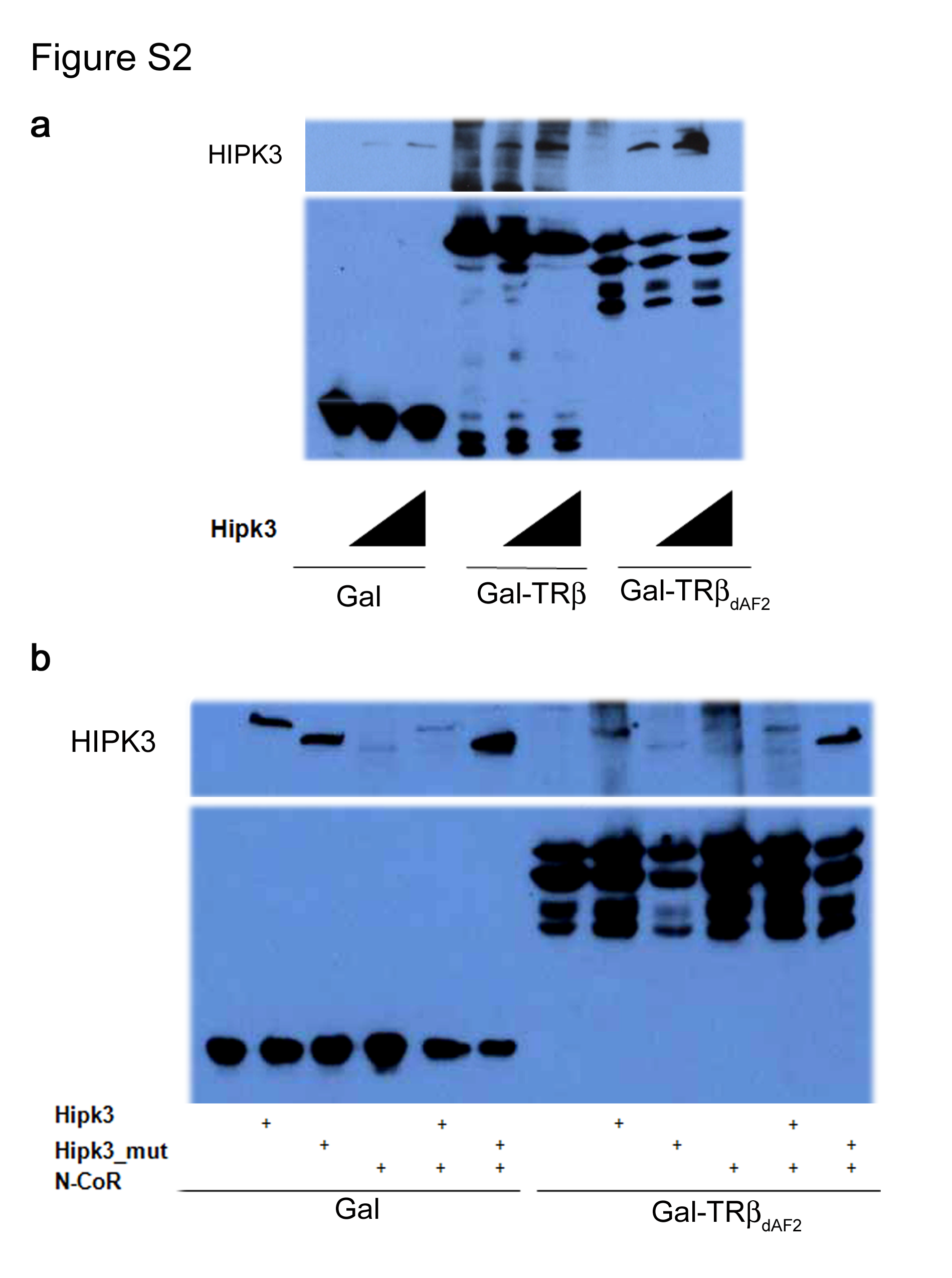

Supplement: Figure S2 — (a/b) Expression of HIPK3 and Gal- and Gal-TR-fusion proteins in reporter assays (Figure 3e and 3f) verified by Western Blot using Flag M2 antibody. (1.62 MB TIF) [file pone.0009797.s002.tif]

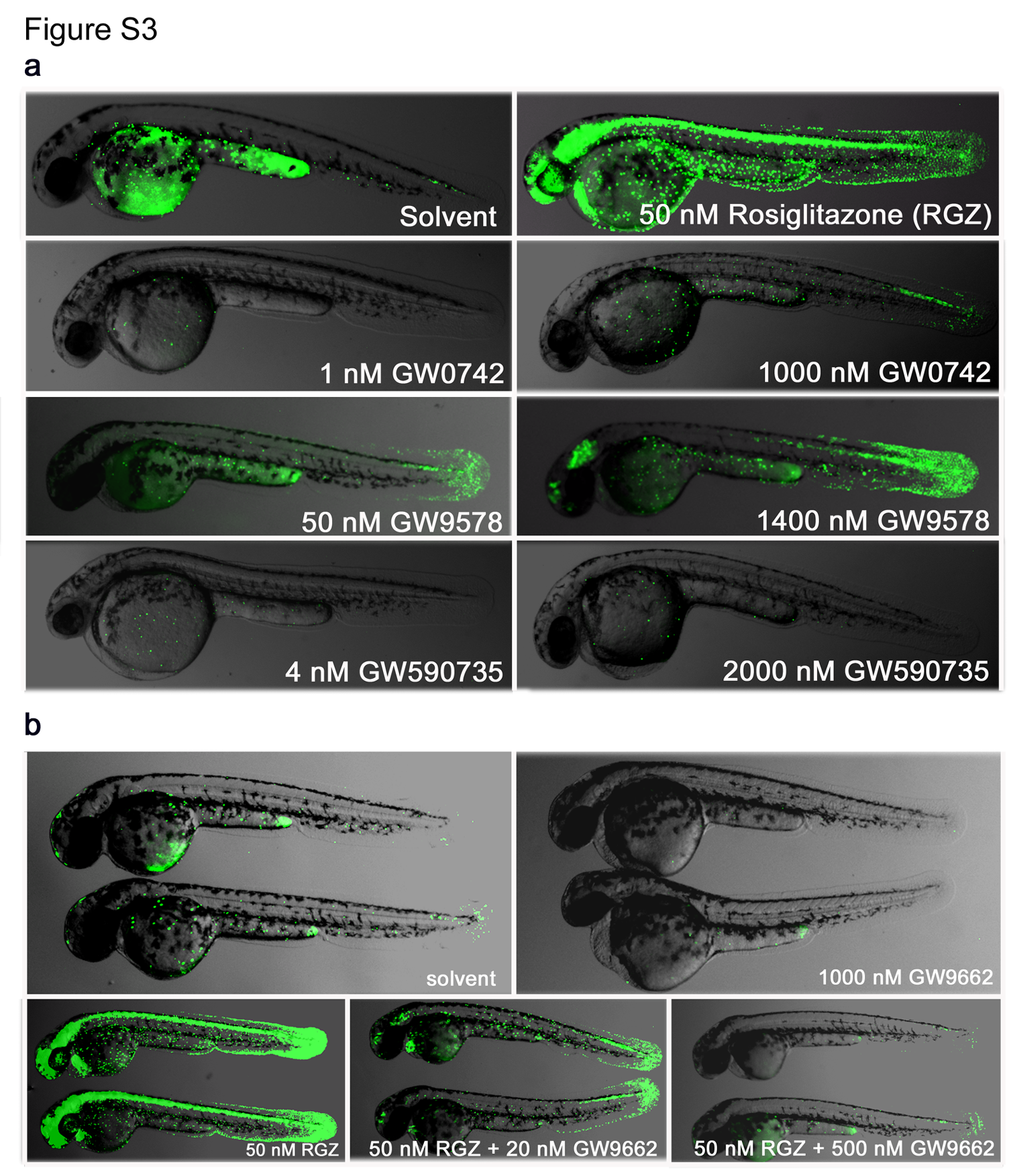

Supplement: Figure S3 — Selective PPARγ drug responses (a) At 24 hpf PPARγ embryos were subjected to a 30 minute heat shock at 37°C and then incubated for 24 hr in the presence of agonists specific for one of the three PPAR isoforms (Rosiglitazone for γ, GW0742 for δ/β and GW9578 or GW590735 for α). The concentrations chosen represent known EC50 values for the appropriate targets, along with significantly higher levels to test for cross-reactivity. Lateral views of 48 hpf embryos, anterior to the left, are shown. (b) PPARγ agonist/antagonist replacement. At 24 hpf PPARγ embryos were subjected to a 30 minute heat induction at 37°C and either incubated for 24 hr in the presence of solvent or 1000 nM GW9662 (upper row) or 50 nM Rosiglitazone (RGZ) alone or 50 nM RGZ and increasing concentrations of GW9662 (20 nM and 500 nM). Two embryos for each treatment showing lateral views at 48 hpf, anterior to the left, are shown. (2.76 MB TIF) [file pone.0009797.s003.tif]
